# Albumin-bound nanoparticle (*nab*) paclitaxel exhibits enhanced paclitaxel tissue distribution and tumor penetration

**DOI:** 10.1007/s00280-015-2833-5

**Published:** 2015-08-01

**Authors:** Nianhang Chen, Carrie Brachmann, Xiping Liu, Daniel W. Pierce, Joyoti Dey, William S. Kerwin, Yan Li, Simon Zhou, Shihe Hou, Michael Carleton, Richard A. Klinghoffer, Maria Palmisano, Rajesh Chopra

**Affiliations:** Celgene Corporation, 86 Morris Avenue, Summit, NJ 07901 USA; Celgene Corporation, San Francisco, CA USA; Presage Biosciences, Seattle, WA USA

**Keywords:** Taxane, Nanoparticle, Albumin, *nab*-paclitaxel, Cremophor EL

## Abstract

**Purpose:**

*nab*-paclitaxel demonstrates improved clinical efficacy compared with conventional Cremophor EL (CrEL)-paclitaxel in multiple tumor types. This study explored the distinctions in drug distribution between *nab*-paclitaxel and CrEL-paclitaxel and the underlying mechanisms.

**Methods:**

Uptake and transcytosis of paclitaxel were analyzed by vascular permeability assay across human endothelial cell monolayers. The tissue penetration of paclitaxel within tumors was evaluated by local injections into tumor xenografts and quantitative image analysis. The distribution profile of paclitaxel in solid-tumor patients was assessed using pharmacokinetic modeling and simulation.

**Results:**

Live imaging demonstrated that albumin and paclitaxel were present in punctae in endothelial cells and could be observed in very close proximity, suggesting cotransport. Uptake and transport of albumin, *nab*-paclitaxel and paclitaxel were inhibited by clinically relevant CrEL concentrations. Further, *nab*-paclitaxel causes greater mitotic arrest in wider area within xenografted tumors than CrEL- or dimethyl sulfoxide-paclitaxel following local microinjection, demonstrating enhanced paclitaxel penetration and uptake by albumin within tumors. Modeling of paclitaxel distribution in patients with solid tumors indicated that *nab*-paclitaxel is more dependent upon transporter-mediated pathways for drug distribution into tissues than CrEL-paclitaxel. The percent dose delivered to tissue via transporter-mediated pathways is predicted to be constant with *nab*-paclitaxel but decrease with increasing CrEL-paclitaxel dose.

**Conclusions:**

Compared with CrEL-paclitaxel, *nab*-paclitaxel demonstrated more efficient transport across endothelial cells, greater penetration and cytotoxic induction in xenograft tumors, and enhanced extravascular distribution in patients that are attributed to carrier-mediated transport. These observations are consistent with the distinct clinical efficacy and toxicity profile of *nab*-paclitaxel.

**Electronic supplementary material:**

The online version of this article (doi:10.1007/s00280-015-2833-5) contains supplementary material, which is available to authorized users.

## Introduction

Paclitaxel is a potent antineoplastic agent with a broad spectrum of activity against solid tumors and is widely used clinically to treat breast, ovarian, lung, prostate and other cancers. It exerts its cytotoxic effects by interfering with microtubule function, leading to altered mitosis and cell death [1, 2]. An obstacle to optimal efficacy of paclitaxel is its hydrophobic nature, which makes it difficult to formulate and deliver. The conventional formulation of paclitaxel requires the drug to be solubilized in the oil-based solvent Cremophor EL (CrEL) and ethanol. However, CrEL-paclitaxel is associated with significant toxicities including severe (sometimes lethal) hypersensitivity reactions (HSRs) and neuropathy in patients. Slow infusion and premedications with corticosteroids and antihistamines are therefore required for CrEL-paclitaxel to prevent HSRs [3–5]. In addition, CrEL alters the disposition of paclitaxel by forming micelles with highly hydrophobic interiors that entrap paclitaxel in circulation, impeding drug delivery to tissue and, consequently, reducing the tumor exposure to paclitaxel [6].

*nab*-Paclitaxel is a novel, solvent-free, 130-nm, albumin-stabilized nanoparticle formulation of paclitaxel. In preclinical studies, *nab*-paclitaxel displayed a higher maximum-tolerated dose, increased antitumor efficacy and prolonged survival compared with solvent-based taxanes (paclitaxel and docetaxel) in mice bearing human tumor xenograft models [7, 8]. Clinically, *nab*-paclitaxel demonstrated superior efficacy and safety to solvent-based taxanes. In a randomized phase 3 study in patients with metastatic breast cancer, *nab*-paclitaxel showed greater efficacy with higher response rates and longer time to tumor progression and a favorable safety profile compared with CrEL-paclitaxel [9]. In this study, patients who had second-line or greater therapy had significantly longer overall survival [9]. In a randomized phase 3 study in patients with non-small cell lung cancer (NSCLC), compared with CrEL-paclitaxel/carboplatin, first-line administration of *nab*-paclitaxel/carboplatin resulted in better tolerability and significantly improved overall response rates in patients with squamous histology [10]. Furthermore, combination of *nab*-paclitaxel and gemcitabine demonstrated significantly longer overall survival and improved clinical outcomes compared with gemcitabine alone in patients with metastatic pancreatic cancer [11], whereas solvent-based taxanes have failed to demonstrate clinically meaningful activity and adequate safety over a series of Phase 2 studies [12–14]. In addition, being solvent-free and devoid of HSRs, *nab*-paclitaxel can be administered to patients at higher doses during a shorter infusion duration and without corticosteroid premedication. Because of the improved benefit/risk ratio, *nab*-paclitaxel has been approved in the USA for the treatment of patients with metastatic breast cancer, locally advanced or metastatic NSCLC, and metastatic adenocarcinoma of the pancreas.

These differences in clinical efficacy/safety between *nab*-paclitaxel and CrEL-paclitaxel are paralleled by significant pharmacokinetic differences [15] with faster, more extensive distribution into the tissue compartments by *nab*-paclitaxel, emphasizing the role of formulation in controlling the disposition of hydrophobic drugs. Previous studies have begun to elucidate the mechanistic basis of these differences. Preclinical results showed that transcytosis of *nab*-paclitaxel across endothelial cell monolayers was increased compared with CrEL-paclitaxel, and *nab*-paclitaxel achieved 33 % higher intratumoral paclitaxel concentration than equal dose of CrEL-paclitaxel in mice bearing human breast tumor xenografts [7]. Clinically, the systemic drug exposure of *nab*-paclitaxel was approximately dose-proportional from 80 to 300 mg/m^2^ and was independent of the intravenous infusion duration [16], whereas CrEL-paclitaxel displays a more than dose-proportional increase in plasma drug exposure and infusion duration-dependent clearance in a manner consistent with increased entrapment in CrEL micelles in circulation with higher dose [17, 18]. In a randomized crossover pharmacokinetic study in patients with solid tumors, the mean fraction of unbound paclitaxel was 2.6-fold higher with *nab*-paclitaxel compared with CrEL-paclitaxel [19], suggesting that CrEL alters drug distribution in blood.

It is hypothesized that *nab*-paclitaxel utilizes the endogenous transport pathways of albumin to achieve enhanced drug delivery and tumor tissue distribution. Albumin has high affinity for hydrophobic drugs including paclitaxel [20] and can be transported across the endothelial barrier of blood vessels through binding to gp60 albumin receptor and activating caveolae-mediated endothelial transcytosis [21–23]. Albumin is highly accumulated in tumors, as tumor cells use albumin as a major energy and nitrogen source through endocytosis and lysosomal degradation [24, 25]. In circulation, *nab*-paclitaxel nanoparticles dissociate in a dynamic process into smaller nanoparticles and eventually to albumin-bound paclitaxel complexes while distributing into tissues. However, the precise mechanism and full effect of albumin-facilitated paclitaxel tumor delivery with *nab*-paclitaxel have yet to be completely elucidated.

The present study was conducted to further characterize drug tissue distribution by *nab*-paclitaxel and investigate the underlying mechanisms. The association of albumin with paclitaxel uptake/transport by human vascular endothelial cells was investigated using in vitro drug transport and imaging assays. The effect of formulation on paclitaxel distribution within human tumor xenografts was measured by evaluating the area and fraction of intratumor mitotic arrest following microinjection into living tumors. Finally, the distribution profile of *nab*-paclitaxel in patients with solid tumors was compared with that of CrEL-paclitaxel using pharmacokinetic modeling and simulation. Taken together, these assessments explain the biological and clinical distinctions between *nab*-paclitaxel and CrEL-paclitaxel.

## Materials and methods

### Reagents and materials

Cremophor EL was obtained from Sigma-Aldrich (Buchs, Switzerland) and EMD Millipore (Billerica, MA, USA). CrEL-paclitaxel was obtained from Teva Pharmaceuticals USA (Sellersville, PA, USA). Paclitaxel and fluorescent-labeled paclitaxel (Flutax-2, Oregon Green 488 conjugated paclitaxel) were obtained from Molecular Probes, Inc (Eugene, OR, USA). *nab*-Paclitaxel (Abraxane^®^) and fluorescent-labeled *nab*-paclitaxel (*nab*-paclitaxel-Flutax, containing 2 % Oregon Green 488 conjugated *nab*-paclitaxel) were manufactured by Celgene Corporation (Summit, NJ, USA). Cell lines of human pancreas carcinoma (MIA PaCa-2, CRL-1420), melanoma (A2058, CRL-11147) and NSCLC (H2122, CRL-5985) were obtained from American Type Culture Collection (ATCC, Manassas, VA, USA). All other reagents and materials were obtained from commercial sources.

### Cellular paclitaxel uptake assay

To evaluate the effect of CrEL on paclitaxel cellular uptake in endothelial cells, 20 μg/mL *nab*-paclitaxel-Flutax was incubated with monolayer HUVECs for 4 h at 37 °C with 5 % CO_2_ in the absence or presence of varying concentrations of CrEL. Cells were trypsinized and washed with phosphate-buffered saline (PBS). The cellular uptake of Flutax was analyzed by FACS.

### In vitro vascular permeability assay

The effect of increasing concentrations of CrEL on paclitaxel transport across endothelial cells was evaluated in an in vitro vascular permeability assay. Briefly, 20 μg/mL of DMSO-dissolved paclitaxel without CrEL (control) or spiked with increasing concentrations of CrEL within clinically relevant range (0.001, 0.01, 0.03, 0.1 and 0.3 %) was added to medium containing 5 % human serum albumin (HSA) above a monolayer of HUVEC cells in a transwell plate at 37 °C with 5 % CO_2_. The medium at the basolateral side was quantitatively analyzed for amount of paclitaxel by LC–MS at indicated time points. The IC_50_ value was estimated by nonlinear fitting of data to a sigmoid model for inhibitory effect, in which the paclitaxel transcytosis was assumed to be 100 % of control with no CrEL and 0 % of control at the infinitively high CrEL concentration.

### Fluorescent microscopy analysis of albumin and paclitaxel uptake by HUVECs

Imaging experiments were performed on early passage HUVEC monolayers in phenol-red-free endothelial basal medium with 2 % FBS and supplements (Lonza). For confocal imaging of albumin uptake, 5 % human albumin was incubated with HUVECs plated on collagen-coated slides for 2–4 h. Slides were moved to ice, and were washed in HBSS, then 2 min in PBS pH 2.6, and several more times in HBSS. Cells were fixed in 4 % formaldehyde/PBS for 10 min at room temperature, permeabilized in 0.1 % saponin/PBS for >1 h and blocked with Odyssey blocking buffer (Li-Cor). Primary antibodies used were: human albumin (H126 Santa Cruz Bio), early endosome antigen 1 (EEA1) or lysosomal-associated membrane protein 1 (LAMP1).

For live widefield microscopy of albumin and fluorescently labeled paclitaxel uptake, early passage HUVEC monolayers on collagen-coated coverslips were incubated for 60 min in 0.005 μg/μL HSA-TRITC alone or with Flutax-2/albumin (Flutax-2 mixed with albumin and diluted to a final concentration of 0.0013 μg/mL Flutax-2). Cells were pre-incubated for 10 min in culture media containing CrEL prior to albumin or HSA-TRITC. Albumin punctae/cell were quantitated by counting 4–5 fields.

### Intratumor paclitaxel pharmacodynamic assay

Animal studies were conducted following all applicable international, national and institutional guidelines for the care and use of animals. To generate xenografts, athymic Nude-Foxn1nu mice (Jackson laboratories) were injected subcutaneously with 2.5 × 10^6^ MIA PaCa-2 cells in a 1:1 ratio with BD Biosciences Matrigel Matrix. Microinjections were performed using the CIVO™ arrayed microinjection device (Presage Biosciences, Seattle, WA, USA) by inserting the device transcutaneously into flank tumors of anesthetized mice. A minimum of three tumors per time point were used with 2–3 replicate injection sites per formulation in each tumor. An average drug volume of 3 μL was delivered via an extrusion method over an injection column length of 6 mm. Inactivated near-infrared dye VivoTag680-S (50 μg/mL) was co-injected with each drug.

At different time points postinjection, animals were euthanized. Tumors were harvested and resected, fixed in 10 % buffered formalin for 48 h, and scanned on a Xenogen IVIS in the near-infrared spectrum (excitation 680 nm, emission 720 nm) to confirm injection sites. Each tumor was cut into 2-mm-thick cross sections perpendicular to the plane of injection to enable a three-dimensional assessment of the entire injection column. Following IVIS imaging, tumors were processed for standard paraffin embedding and histological analysis. Four micron sections cut from each 2 mm gross level as described above were stained with anti-phospho-histone H3 (pHH3) antibody and Alexa Fluor 488 secondary antibody to assess drug-induced tumor responses (mitotic arrest) using custom software (CIVOanalyzer™; Presage Biosciences, Seattle). Mean fraction values of pHH3 positive cells were plotted with standard error bars, as a function of radial distance for each formulation and time point. To assess the statistical significance of differences between any pair of formulations, a linear mixed model approach was used. In the model, the response to the CrEL-paclitaxel formulation was assumed to be a random effect, and the differential response due to *nab*-paclitaxel or DMSO-paclitaxel was assumed to be fixed effects. A *p* value <0.05, adjusted for multiple comparisons, indicates statistically significant differences.

### Pharmacokinetic simulations of *nab*-paclitaxel and CrEL-paclitaxel

The population pharmacokinetic model of *nab*-paclitaxel was described previously [26]. The analysis dataset included 150 patients enrolled in 8 clinical studies. The studies were conducted in accordance with the ethical principles originating in the Declaration of Helsinki and ICH Good Clinical Practice guidelines, applicable regulatory requirements, and in compliance with the protocols. All patients provided written informed consent.

All patients had advanced or metastatic solid tumors. *nab*-Paclitaxel was administered intravenously as monotherapy over the dose range of 80–375 mg/m^2^. Paclitaxel concentrations in whole blood or plasma were measured at specified time points for up to 72 h postdose. The population pharmacokinetic model of CrEL-paclitaxel was developed by Joerger et al. [27] using similar methodology. The CrEL-paclitaxel analysis dataset included 168 solid-tumor patients enrolled in five clinical studies. CrEL-paclitaxel was administered intravenously over the dose range of 100–250 mg/m^2^, with plasma concentration of paclitaxel measured up to 48 h postdose. The distribution of patient demographics (age, gender, body surface area) and baseline parameters associated with hepatic or renal function were comparable between the two analysis datasets (20, 27).

The paclitaxel exposure in plasma (the central compartment) and peripheral tissues/organs (the first and second peripheral compartments) was compared between *nab*-paclitaxel and CrEL-paclitaxel using simulations. The concentration–time profile in the central and peripheral compartments for a “typical” patient in the respective analysis dataset (median values for each of the covariates included in the final model) was simulated using the published typical model parameters (population estimates) for the following three scenarios: (1) at the approved maximum dose for the once every 3 week (Q3W) dosing schedule, which is 260 mg/m^2^ over 0.5-h infusion for *nab*-paclitaxel and 175 mg/m^2^ over 3-h infusion for CrEL-paclitaxel; (2) at the commonly used dose for the once weekly (QW) dosing schedule, which is 100 mg/m^2^ over 0.5-h infusion for *nab*-paclitaxel and 80 mg/m^2^ over 1-h infusion for CrEL-paclitaxel; and (3) at the same infusion duration of 1-h over the clinical dose range of 80 to 300 mg/m^2^ for both formulations. The amount of drug in each peripheral compartment was estimated by multiplying the simulated drug concentration in the peripheral compartment with the volume of distribution corresponding to the compartment.

## Results

### Uptake and transport of paclitaxel is facilitated by albumin and inhibited by Cremophor EL

**Fig. 1:**
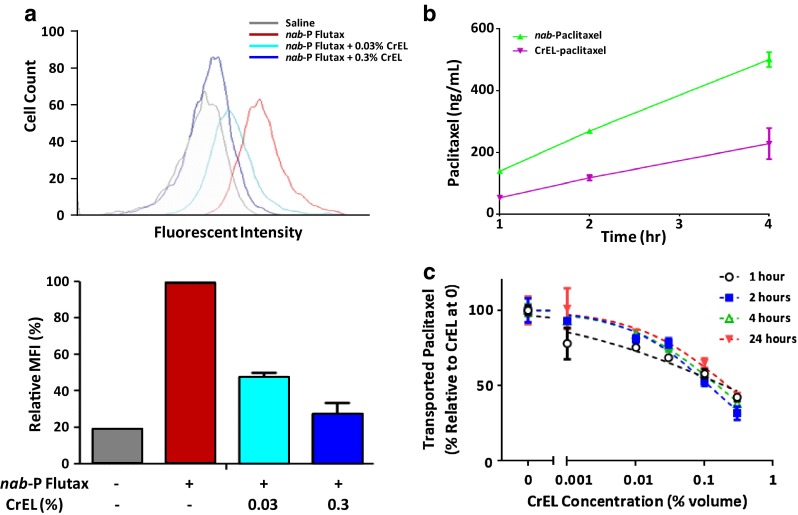
Cremophor-EL-inhibited paclitaxel cellular uptake and transport across endothelial cell monolayer. **a** The effect of CrEL on uptake of *nab*-paclitaxel-Flutax by HUVECs was evaluated in a flow cytometry-based assay. The *upper panel* shows the FACS intensity distributions in the Flutax channel, and the *lower panel* shows the derived mean fluorescence intensity (MFI) values. **b** Paclitaxel transport across intact monolayers of HUVEC cells with *nab*-paclitaxel and CrEL-paclitaxel. **c** Inhibition of paclitaxel transport across intact monolayers of HUVEC cells by CrEL at 1, 2, 4, and 24 h. The amount of paclitaxel transcytosis at different CrEL concentrations, calculated relative to paclitaxel transport in the absence of CrEL, is plotted. The *dashed lines* show fit to a sigmoid model for inhibitory effect, in which the paclitaxel transcytosis was assumed to be 100 % of control with no CrEL and the baseline (maximally inhibited value) was fixed at 0 % of control

In a previous study, it has been shown that increasing concentrations of CrEL can inhibit binding of paclitaxel to human serum albumin and human umbilical vein endothelial cells (HUVECs) in a dose-dependent manner [7]. In this study, the effect of CrEL on uptake of *nab*-paclitaxel into endothelial cells was evaluated in a fluorescence-activated cell sorting (FACS) assay using fluorescent-labeled *nab*-paclitaxel (*nab*-paclitaxel-Flutax). Results showed that *nab*-paclitaxel-Flutax uptake by HUVECs was strongly inhibited with increasing concentrations of CrEL (Fig. 1a). HUVECs incubated with *nab*-paclitaxel-Flutax had high fluorescence intensity, demonstrating efficient cellular uptake. In contrast, CrEL at a clinically relevant concentration of 0.3 % [28] almost completely inhibited cellular uptake of *nab*-paclitaxel-Flutax, and reduced fluorescence levels close to those of unstained cells.

To model the effect of CrEL on extravasation of paclitaxel, drug transport across intact endothelial cell monolayers was determined using unlabeled *nab*-paclitaxel and CrEL-paclitaxel. Drugs were added to the media above a monolayer of HUVECs in a transwell plate, and the level of paclitaxel transported across the HUVEC monolayer was determined by liquid chromatography–mass spectrometry (LC–MS). The amount of paclitaxel transport across endothelial cells was significantly higher with *nab*-paclitaxel than CrEL-paclitaxel (Fig. 1b). In addition, CrEL strongly inhibited paclitaxel transport across endothelial cells, with fit-determined concentrations required for 50 % inhibition of paclitaxel transcytosis (IC_50_) of 0.19, 0.12, 0.16 and 0.22 % at 1, 2, 4 and 24 h, respectively (Fig. 1c).

### Localization of albumin and paclitaxel in endosomal vesicles of endothelial cells

**Fig. 2:**
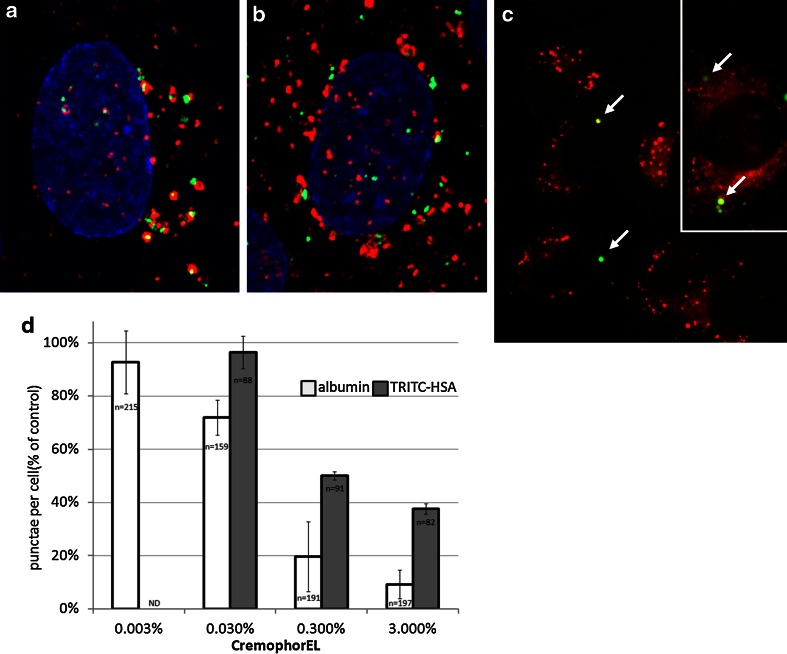
Endocytosed albumin in endothelial cells was visualized in endocytic vesicles, in close proximity to vesicular paclitaxel. **a** Albumin (*green*) was found in endocytic vesicles, some of which were early endosomes as indicated by colocalization with EEA1 (*red*). **b** Very little albumin (*green*) colocalized with the lysosomal marker, LAMP1 (*red*). **a**, **b** Are digital close-ups of a representative confocal image with nuclei shown in *blue*. **c** Merged image of live visualization of albumin (HSA-TRITC in *red*) and Flutax-2/albumin (*green*) uptake. *White arrows* indicate vesicles containing both fluorescent molecules. *Inset* shows image of unusually large HUVEC cell with a large number of fluorescent punctae. **d** Increasing concentrations of CrEL-inhibited endocytosis of albumin. Endothelial cells were incubated with albumin or HSA-TRITC and CrEL for 2 h and the number of punctae per cell are graphed. The number of cells counted for each condition is indicated

In a previous study, albumin internalized by monolayer endothelial cells was found in plasmalemmal vesicles but not in lysosomes [23]. To further determine the mechanism and the role of albumin in paclitaxel uptake by endothelial cells, monolayer HUVECs were incubated with human albumin. Consistent with the previous study, internalized albumin was observed in endocytic vesicles, some of which were early endosomes as indicated by the presence of EEA1 protein (Fig. 2a). Additionally, very little albumin was found in lysosomes, as indicated by the staining of LAMP1 (Fig. 2b). These findings are consistent with endocytic uptake of albumin and transendothelial trafficking of the molecule rather than breakdown of the protein in lysosomes. To determine whether albumin-associated paclitaxel could also be visualized in vesicles, monolayer HUVECs were incubated with fluorescently labeled paclitaxel (Flutax-2) mixed with albumin and fluorescently labeled albumin (HSA-TRITC). Live imaging demonstrated that both fluorescent molecules were present in punctae and could be observed in very close proximity (Fig. 2c). Consistent with vesicle trafficking, no fluorescent paclitaxel was found in lysosomes as visualized by LysotrackerRed (data not shown). The combined results demonstrate that paclitaxel can be found in punctae in endothelial cells, and that their pattern and proximity to albumin-containing vesicles suggests that paclitaxel utilizes the same endocytosis and transcytosis mechanism as albumin. To investigate whether CrEL influences albumin uptake, endothelial cells were incubated with albumin in the presence of increasing concentrations of CrEL and the number of albumin punctae was quantified by microscopic imaging. Consistent with FACS assay results of paclitaxel uptake, a concentration-dependent decrease in albumin uptake in the presence of clinically relevant concentrations (up to 0.3 %) of CrEL was observed with both anti-albumin immunofluorescent staining and HSA-TRITC (Fig. 2d).

### Paclitaxel penetration within tumors facilitated by albumin but limited by solvents

**Fig. 3:**
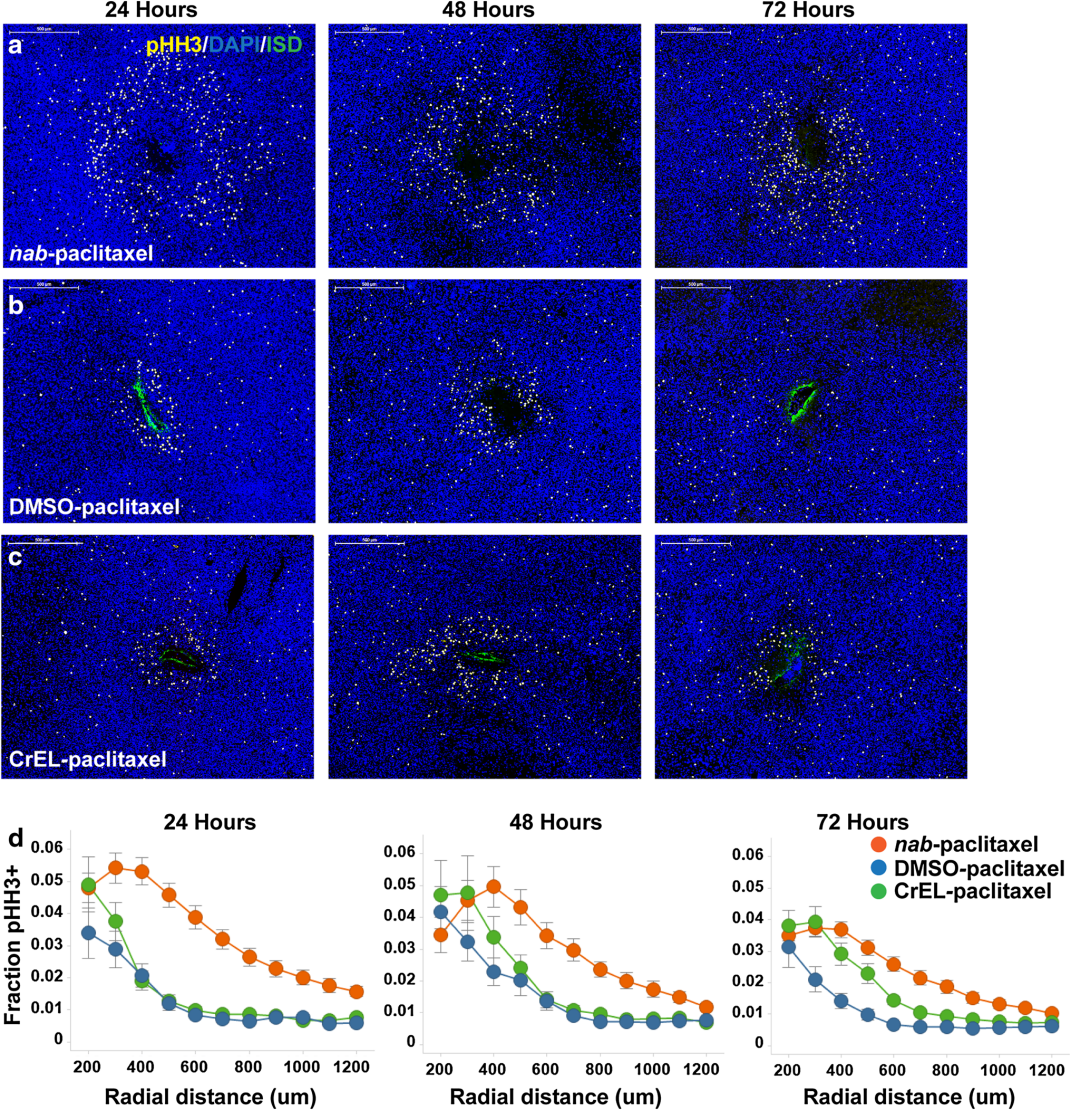
*nab*-Paclitaxel induced increased mitotic arrest in a larger area within MIA PaCa-2 tumor xenografts compared with solvent-based paclitaxel. **a**–**c** Representative immunohistochemical images of mitotically arrested cells in pancreatic MIA PaCa-2 xenograft tumors at 24, 48 and 72 h following microinjection with equal amounts (12 μg) of *nab*-paclitaxel (**a**), DMSO-paclitaxel (**b**) and CrEL-paclitaxel (**c**). Each drug was co-injected with an inert near-infrared dye (*green*) to delineate injection sites. Mitotically arrested cells were stained with anti-pHH3 antibody (*white*) and nuclei were stained with DAPI (*blue*). Representative images from a single injection site are shown. Immunohistochemical analysis for pHH3 shows responses to each formulation extending radially from the* center* of the injection site. *Scale bars* 500 μm. **d** Fraction of arrested cells as a function of distance from the center of the injection site in pancreatic MIA PaCa-2 xenograft tumors (*n* = 8; *p* < 0.01) at 24, 48 and 72 h postinjection, respectively. In* each row* (**a**–**d**), the *left*, *middle* and *right panels* show the results at 24, 48 and 72 h after injection, respectively. Data are expressed as mean ± standard error

To assess whether paclitaxel formulations measurably impact drug penetration through tumor tissue and uptake into target cells, a novel and highly precise instrument was used to simultaneously microinject multiple paclitaxel formulations into different regions of the same tumor, facilitating subsequent quantitative comparisons. Equal amounts of *nab*-paclitaxel, CrEL-paclitaxel and DMSO-paclitaxel, as verified by LC–MS (data not shown), were delivered through direct intratumoral microinjection into flank human pancreatic MIA PaCa-2 tumor xenografts. Tumors were analyzed 24, 48 or 72 h postdrug microinjection for mitotic arrest by immunofluorescent staining of phospho-histone H3 (pHH3), which was used as a pharmacodynamic indicator of paclitaxel activity to monitor drug penetration and tumor cell uptake at defined radial distances extending from the site of injection. Exposure to all three formulations of paclitaxel induced an increase in the number of pHH3-positive cells, which diminished with further radial distance from the site of injection (Fig. 3a–d). Importantly, at all three time points, the area of response and the total fraction of pHH3-positive cells at a specific radial distance were significantly greater for microinjected *nab*-paclitaxel compared with either of the solvent-based CrEL-paclitaxel and DMSO-paclitaxel (*p* < 0.01) (Fig. 3a–d).

Similarly, microinjected *nab*-paclitaxel induced a larger increase in both the area of response and total fraction of cells arrested in mitosis at 24 h postinjection when compared to CrEL-paclitaxel-injected A2058 melanoma (*n* = 5 tumors; *p* < 0.001) and DMSO-paclitaxel-injected H2122 NSCLC xenografts (*n* = 3 tumors; *p* < 0.001) (Supplement Fig. 1).

### Enhanced paclitaxel distribution to tissues by *nab*-paclitaxel in patients with solid tumors mainly attributable to a saturable transport process

**Fig. 4:**
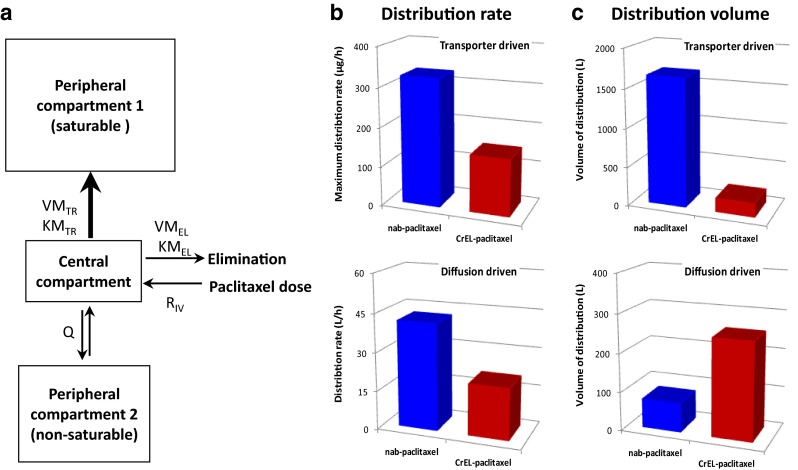
Pharmacokinetic model and parameters describing paclitaxel distribution with *nab*-paclitaxel and CrEL-paclitaxel in patients with solid tumors. **a** Three-compartment PK model and parameters. KM_EL_ = paclitaxel plasma concentration at half VM_EL_; KM_TR_ = paclitaxel plasma concentration at half VM_TR_; Q = intercompartmental clearance between the central and second peripheral compartment; R_IV_ = infusion rate; VM_EL_ = maximum elimination rate; VM_TR_ = maximum distribution rate from the central to the first peripheral compartment. **b** Distribute rate through transporter-driven and passive diffusion-driven distribution for *nab*-paclitaxel and CrEL-paclitaxel. **c** Distribute volume through transporter-driven and passive diffusion-driven distribution for *nab*-paclitaxel and CrEL-paclitaxel

The plasma concentration versus time data of paclitaxel in solid-tumor patients treated with *nab*-paclitaxel or CrEL-paclitaxel were best described by a three-compartment pharmacokinetic model [26, 27]: the central compartment (plasma and well perfused organs), the first peripheral compartment (tissues/organs to which the drug was distributed through a saturable transporter-mediated mechanism) and the second peripheral compartment (tissue/organs to which the drug was distributed through a non-saturable passive diffusion) (Fig. 4a). The rate of both saturable transporter-driven and passive distribution is more than doubled with *nab*-paclitaxel versus CrEL-paclitaxel (Fig. 4b), consistent with previous data showing faster distribution of drug into tissues with *nab*-paclitaxel [26]. The volume of the first peripheral compartment involving saturable distribution of paclitaxel (Fig. 4c) was approximately ninefold larger when administered as *nab*-paclitaxel (1650 L) versus CrEL-paclitaxel (177 L), consistent with deeper penetration of the drug into tissues via transporter-mediated pathways with *nab*-paclitaxel. In contrast, the volume of distribution of the second peripheral compartment involving passive diffusion (Fig. 4c) was approximately 70 % smaller for *nab*-paclitaxel (75.4 L) versus CrEL-paclitaxel (252 L).

**Fig. 5:**
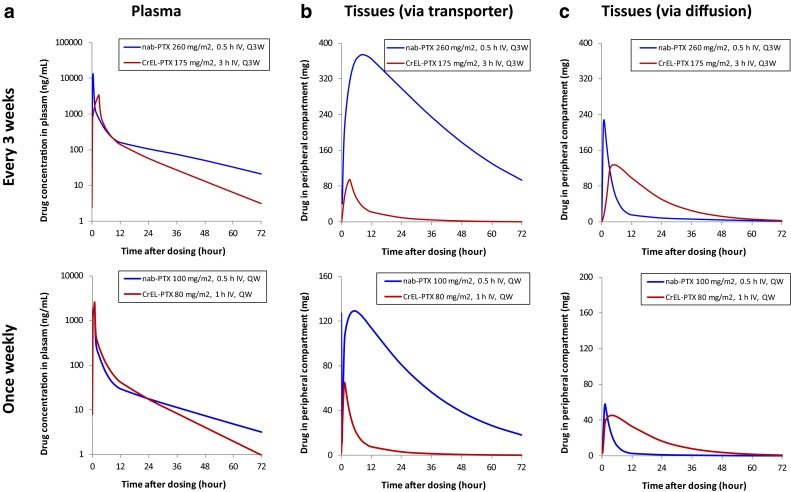
Model-predicted drug exposure in plasma and peripheral tissues at commonly used clinical doses for *nab*-paclitaxel and CrEL-paclitaxel in patients with solid tumors. **a** Predicted drug exposure versus time plot for plasma with *nab*-paclitaxel and CrEL-paclitaxel at Q3W and once weekly dosing schedules. **b** Predicted tissues distribution via transporter with *nab*-paclitaxel and CrEL-paclitaxel at Q3W and once weekly dosing schedules. **c** Predicted tissue distribution via diffusion with *nab*-paclitaxel and CrEL-paclitaxel at Q3W and once weekly dosing schedules. PTX = paclitaxel; Q3W = once every 3 weeks; QW = once every week

The model-predicted drug concentrations in plasma were compared between *nab*-paclitaxel and CrEL-paclitaxel at the therapeutic dose levels for the Q3W and the QW dosing schedules, respectively (Fig. 5a). The difference in the predicted plasma concentration profiles was relatively small between the two formulations, especially for the QW dosing regimens. The area under the plasma concentration–time curve (AUC) was estimated to be similar between *nab*-paclitaxel 100 mg/m^2^ over 0.5-h infusion (3875 h ng/mL) and CrEL-paclitaxel 80 mg/m^2^ over 1-h infusion (4120 h ng/mL).

The model-predicted drug exposure in the two peripheral compartments were also compared between *nab*-paclitaxel and CrEL-paclitaxel at therapeutic dosing regimens. At the approved maximum Q3W dose for the treatment of breast cancer, the model predicted that *nab*-paclitaxel (260 mg/m^2^ over 0.5-h infusion) would deliver considerably more drugs into tissues via transporter-mediated pathways than CrEL-paclitaxel (175 mg/m^2^ over 3-h infusion) (Fig. 5b). In contrast, CrEL-paclitaxel was predicted to deliver drugs into tissues mainly via passive diffusion (Fig. 5c). In recent years, weekly CrEL-paclitaxel (with a lower dose and shorter infusion duration) has been considered as a more effective and less toxic dosing regimen than the Q3W dosing regimen [29, 30]. For the weekly dosing regimen, *nab*-paclitaxel (100 mg/m^2^ over 0.5-h infusion) was predicted to deliver considerably more drugs into the tissues via transporter-mediated pathways than CrEL-paclitaxel (80 mg/m^2^ over 1-h infusion) (Fig. 5b), even though their plasma AUC was similar (Fig. 5a).

**Fig. 6:**
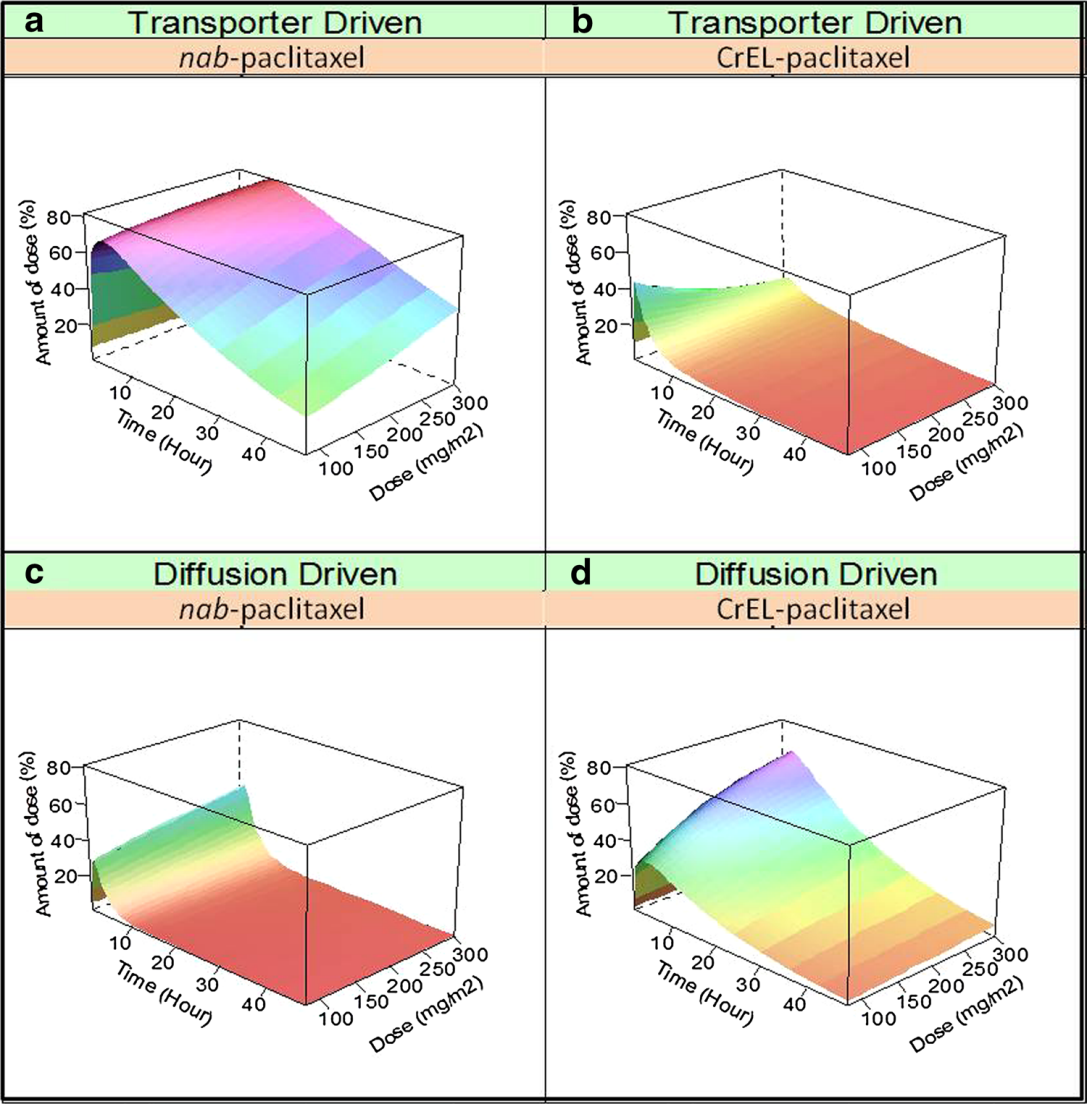
Model-predicted effect of dose on drug distribution in peripheral tissues with the same infusion duration for *nab*-paclitaxel and CrEL-paclitaxel in patients with solid tumors. **a**, **b** Contribution of transporter-driven drug distribution relative to dose of *nab*-paclitaxel and CrEL-paclitaxel. **c**, **d** Contribution of diffusion-driven drug distribution relative to dose of *nab*-paclitaxel and CrEL-paclitaxel

The relationship between dose and distribution in tissues was further assessed by assuming the same infusion duration (1 h) for both formulations, with the exposure in a given peripheral compartment being expressed as percentage of the administered dose (Fig. 6). Regardless of dose levels, a larger percentage of the administered dose was predicted to distribute into tissues via transporter-mediated pathways with *nab*-paclitaxel (Fig. 6a) than with CrEL-paclitaxel (Fig. 6b) at all tested postdosing time points. For *nab*-paclitaxel, the maximum amount of drug in the first peripheral compartment would account for approximately 70 % of the administered dose at 80 mg/m^2^, and the maximum percent distribution would remain constant or slightly higher (up to 81 % of the dose) when increasing the dose level from 80 to 300 mg/m^2^ (Fig. 6a). For CrEL-paclitaxel, the maximum amount of drug in the first peripheral compartment would account only for approximately 45 % of the administered dose at 80 mg/m^2^, and the maximum percent distribution would decrease to 19 % with increasing dose to 300 mg/m^2^ (Fig. 6b). On the other hand, with increasing dose from 80 to 300 mg/m^2^, the maximum passive diffusion would increase from 31 to 65 % of the dose for CrEL-paclitaxel (Fig. 6d) but only from 31 to 44 % of the dose for *nab*-paclitaxel (Fig. 6c).

## Discussion

The present study demonstrated that *nab*-paclitaxel nanoparticles and albumin-bound paclitaxel can utilize biological albumin pathways, transport across endothelial cell layers, penetrate through tumor tissue, and disrupt mitotic progression more effectively within tumors, whereas solvents (CrEL or DMSO) strongly restricted these processes. Further, a pharmacokinetic model developed based on clinical data illustrated the relative contribution of transporter-mediated distribution and passive diffusion on the tissue distribution of *nab*-paclitaxel and CrEL-paclitaxel, providing a rationale for the clinical advantages of *nab*-paclitaxel.

A series of in vitro assays with HUVECs measured cellular uptake of paclitaxel and albumin by FACS and microscopic imaging, and paclitaxel transcytosis by in vitro vascular permeability assay. Importantly, albumin and paclitaxel could be found in punctae that were in very close proximity, suggesting that they were in the same endosomal vesicles. Results of these orthogonal tests consistently demonstrated that *nab*-paclitaxel nanoparticles and albumin-bound paclitaxel can utilize the endogenous albumin pathways to transport across the endothelial barrier of blood vessels via a non-lysosomal endocytosis process. Transendothelial cell transport of albumin is proposed to be mediated by the gp60 (albondin) receptor, which is located on the endothelial cell surface and binds to albumin with a high affinity in the nanomolar range [21]. Albumin binding to gp60 activates caveolin-1 and induces the formation of caveolae, which transport albumin and other plasma constituents across the endothelial cell to the interstitial space [31]. This active transport mechanism is consistent with rapid distribution of *nab*-paclitaxel from the central compartment as shown in our pharmacokinetic model.

Our in vitro study results explain why CrEL-paclitaxel, unlike *nab*-paclitaxel, cannot efficiently exploit the biological albumin transport mechanism. It was reported previously that the binding of paclitaxel to human serum albumin was inhibited by CrEL in a dose-dependent manner, possibly secondary to micelle sequestration of paclitaxel [7]. In this study, the uptake of both albumin and *nab*-paclitaxel was similarly inhibited by the presence of CrEL, suggesting for the first time that CrEL may reduce paclitaxel transport via inhibiting albumin transport. This is an effect different from the conventional concept of micellar entrapment [6], as both albumin and *nab*-paclitaxel particles are hydrophilic and thus not expected to partition into the highly hydrophobic interior of CrEL micelles. In addition, our study extended the previous findings using fluorescent labels as a surrogate marker for paclitaxel transcytosis [7] by demonstrating transcytosis of unlabeled paclitaxel, a process facilitated by albumin and *nab*-paclitaxel but inhibited by CrEL. Entrapment of paclitaxel molecules in CrEL micelles may partially inhibit paclitaxel transcytosis as the critical micellar concentration (CMC) of CrEL is 0.009 % in aqueous solution [32]. The peak plasma CrEL level achieved after intravenous administration of therapeutic doses (100–175 mg/m^2^) of CrEL-paclitaxel over a 3-h period is approximately 0.3–0.5 % [28], and the plasma CrEL level at 24 h following infusion is in the range of 0.1 % [33]. Thus, the CrEL concentrations at 0.3 % and below tested in this study are clinically relevant.

The volume of distribution of CrEL in humans is extremely low and not much higher than the blood volume, implying limited tissue and tumor delivery of CrEL [34]. The inhibition of innate albumin transport pathways and/or micellar sequestration by CrEL results in the prolonged retention of paclitaxel in plasma and retardation of tissue distribution. The inhibitory effect by CrEL is concentration-dependent; therefore, the impact of CrEL on extravascular distribution of paclitaxel becomes even more pronounced at higher doses of CrEL-paclitaxel, which is predicted by our model and consistent with the nonlinear dose–exposure relationship of CrEL-paclitaxel observed in patients. At higher doses of CrEL, the percentage of paclitaxel trapped in plasma increases disproportionately [34, 35] and CrEL-paclitaxel relies more on passive diffusion for down-hill distribution into tissues.

Another key advantage conferred by *nab*-paclitaxel identified in the present study is that albumin-bound paclitaxel complexes can distribute effectively and extensively within tumors, whereas solvents such as CrEL and DMSO severely limit intratumor drug distribution. The controlled localization of treatments delivered with the Presage platform bypasses vasculature-dependent drug delivery, allowing direct assessment of tumor tissue penetration and target cell uptake across the span of a living tumor while capturing the heterogeneity of cancer cells. In our study, *nab*-paclitaxel caused high levels of mitotic arrest over a larger area after local injection into tumors than CrEL-paclitaxel and DMSO-paclitaxel, strongly supporting albumin-mediated enhancement of paclitaxel tissue penetration and tumor cell uptake, which likely contributes to the increased antitumor effect of *nab*-paclitaxel over solvent-based paclitaxel in preclinical and clinical studies. There are several major hurdles for intratumor drug distribution, including high interstitial fluid pressure, desmoplastic structures such as collagen fiber networks, and high density growth of cancer cells [36–38]. Proliferating tumor cells actively take up albumin via endocytosis where it is catabolized, and the derived amino acids are used for de novo protein synthesis, energy and growth [24, 25]. As such, *nab*-paclitaxel may enable the drug to be delivered to tumor tissues that are less accessible to CrEL-paclitaxel, thereby improving the efficacy for certain tumors and being effective in tumors unresponsive to CrEL-paclitaxel. This distinction may be particularly critical for patients with desmoplastic tumors such as pancreatic cancer, which pose major challenges for drug delivery. Indeed, in patients with advanced pancreatic cancer, *nab*-paclitaxel is the only taxane formulation that has demonstrated clinically significant improvement in overall survival when administered in combination with gemcitabine [11]. Combination treatment with *nab*-paclitaxel increases intratumoral gemcitabine levels in mouse models of pancreatic cancer, which has been attributed to stromal disrupting effects of *nab*-paclitaxel [39], or a marked decrease in the levels of cytidine deaminase, the primary gemcitabine metabolizing enzyme [40].

We further analyzed the roles played by the formulation in tissue distribution of *nab*-paclitaxel and CrEL-paclitaxel using a pharmacokinetic model. The common model structure provided a consistent framework to compare the drug distribution kinetics for *nab*-paclitaxel with that reported for CrEL-paclitaxel in published literature [27]. Saturable kinetics for distribution and elimination have long been described for CrEL-paclitaxel [27, 41] and have been attributed to entrapment of paclitaxel in CrEL micelles [6]. However, the identification of saturable distribution and elimination kinetics for *nab*-paclitaxel indicates that saturable kinetics is probably also due to saturable transport processes. The pharmacokinetic model reveals distinct distribution mechanisms for *nab*-paclitaxel and CrEL-paclitaxel in patients with solid tumors. The distribution of *nab*-paclitaxel is more dependent upon transporter-mediated pathways, reflected as a faster rate and a larger volume for saturable drug distribution to the first peripheral compartment compared to CrEL-paclitaxel. Conversely, drug delivery into tissue by CrEL-paclitaxel is more dependent upon passive diffusion. Moreover, the fraction of *nab*-paclitaxel dose delivered to tissues would remain relatively constant for either transporter-mediated or diffusion-related distribution over a broad clinical dose range. In contrast, transporter-mediated distribution decreases, while diffusion-related distribution increases with higher dose of CrEL-paclitaxel. These findings are consistent with the notion that *nab*-paclitaxel facilitates the drug distribution by exploiting the physiological transporter properties of albumin and can explain why at commonly used clinical doses, *nab*-paclitaxel would deliver more active drugs to the tumor.

Understanding of drug distribution mechanisms will enable improved dosing regimens for *nab*-paclitaxel and CrEL-paclitaxel. *nab*-Paclitaxel is predicted to have a relatively stable distribution pattern between the two peripheral compartments regardless of the dose level, allowing a more predictable efficacy/safety profile upon dose escalation or when switching dosing schedules. However, the dose-dependent tendency toward diffusion-driven drug distribution for CrEL-paclitaxel might lead to redistribution of the drug when changing the dosing regimen with the potential to adversely affect clinical outcomes.

For drugs such as *nab*-paclitaxel and CrEL-paclitaxel where the drug concentration or AUC in systemic circulation is driven largely by distribution rather than elimination and the drug target (for solid tumors) is outside systemic circulation, the systemic drug concentration or AUC may not be a good surrogate for their pharmacologic effect. Rather, the distribution of drug into tissues should be a better predictor of associated drug effect. The simulation results suggested that even at the same dose level (or comparable plasma AUC), the drug exposure profile in the peripheral compartments would differ remarkably between *nab*-paclitaxel and CrEL-paclitaxel, which is expected to result in distinct safety and efficacy profile in patients. Indeed, different efficacy and tolerability for *nab*-paclitaxel and CrEL-paclitaxel were observed in clinical studies with breast [9] and lung cancer [10]. One potential explanation for the enhanced tissue distribution but improved tolerability for *nab*-paclitaxel is that paclitaxel exposure in normal tissues is reduced whereas more paclitaxel is delivered into tumor via albumin transport pathways, as demonstrated by results in preclinical tumor xenograft models [7, 42]. In addition, the faster tissue distribution by *nab*-paclitaxel causes a shorter duration of high drug concentrations in plasma, which has been found to reduce the risk of the dose-limiting toxicity neutropenia [26]. In contrast, the retention of paclitaxel by CrEL in circulation prolongs systemic drug exposure, resulting in higher risks of neutropenia [34].

In addition to *nab*-paclitaxel and CrEL-paclitaxel, the distribution kinetics of another taxane, docetaxel, was examined based on the population pharmacokinetic model developed by Bruno et al. [43]. The approved maximum doses of the three taxanes (*nab*-paclitaxel, CrEL-paclitaxel and docetaxel) for the Q3W dosing schedule produced distinct delivery efficiency of taxane payload in descending order of *nab*-paclitaxel, CrEL-paclitaxel and docetaxel (data not shown). The low tissue distribution of docetaxel might in part account for its checkered efficacy and safety compared to CrEL-paclitaxel in spite of its higher in vitro antitumor potency [44, 45].

In conclusion, compared with the solvent-based paclitaxel, *nab*-paclitaxel demonstrates more efficient transport across endothelial cells, greater penetration, cell uptake and mitotic arrest induction in tumor xenografts, and enhanced extravascular distribution in patients that are attributable to carrier-mediated transport. These findings highlight the advantage of *nab*-paclitaxel in drug delivery to tissues and targets, and provide mechanistic insight into distinctions between the efficacy and safety profiles of *nab*-paclitaxel and solvent-based paclitaxel in the treatment of patients with solid tumors.

## Electronic supplementary material


**Supplement Fig.** **1** *nab*-Paclitaxel induced increased mitotic arrest in a larger area within A2058 and H2122 tumor xenografts compared with solvent-based paclitaxel. (a) Fraction of mitotically arrested cells as a function of distance from the center of the injection site in A2058 melanoma xenografts (*n* = 5; *p* < 0.001) at 24 h postinjection. (b) Fraction of mitotically arrested cells as a function of distance from the center of the injection site in H2122 NSCLC xenografts (*n* = 3; *p* < 0.001) at 24 h postinjection. Data are expressed as mean ± standard error (TIFF 2140 kb)
