# Supplementary figures and images for: Albumin-bound nanoparticle (nab) paclitaxel exhibits enhanced paclitaxel tissue distribution and tumor penetration
Source: Cancer Chemother Pharmacol. 2015 Aug 1;76:699–712. doi: 10.1007/s00280-015-2833-5 (PMC4768222; doi:10.1007/s00280-015-2833-5)

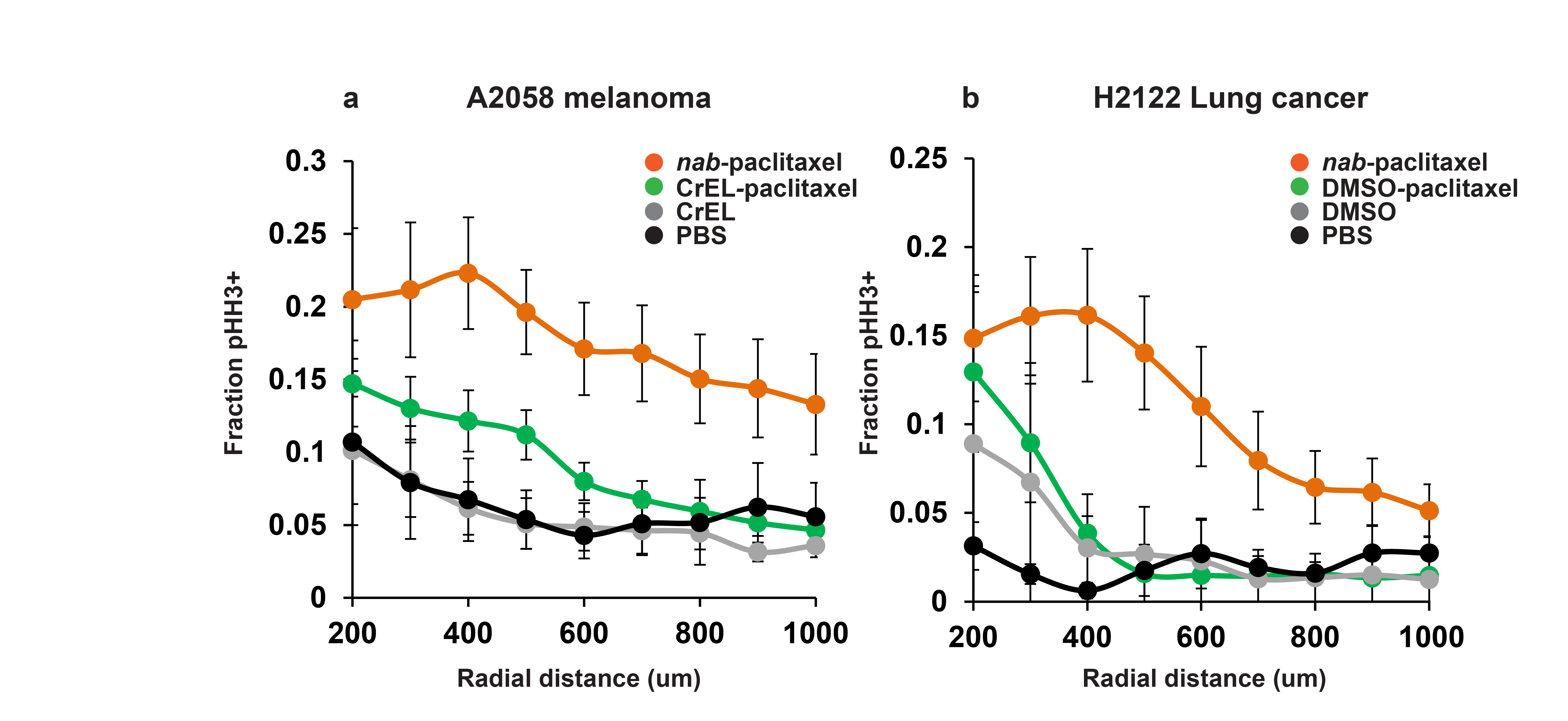

Supplement: Supplementary file 1 — Supplement Fig. 1 nab-Paclitaxel induced increased mitotic arrest in a larger area within A2058 and H2122 tumor xenografts compared with solvent-based paclitaxel. (a) Fraction of mitotically arrested cells as a function of distance from the center of the injection site in A2058 melanoma xenografts (n = 5; p < 0.001) at 24 h postinjection. (b) Fraction of mitotically arrested cells as a function of distance from the center of the injection site in H2122 NSCLC xenografts (n = 3; p < 0.001) at 24 h postinjection. Data are expressed as mean ± standard error (TIFF 2140 kb) [file 280_2015_2833_MOESM1_ESM.tif]
